# Author Correction: Jawbone microenvironment promotes periodontium regeneration by regulating the function of periodontal ligament stem cells

**DOI:** 10.1038/s41598-020-65652-3

**Published:** 2020-05-27

**Authors:** Bin Zhu, Wenjia Liu, Yihan Liu, Xicong Zhao, Hao Zhang, Zhuojing Luo, Yan Jin

**Affiliations:** 1 State Key Laboratory of Military Stomatology, Centre for Tissue Engineering, School of Stomatology, Fourth Military Medical University, Xi’an, Shaanxi People’s Republic of China; 2 Department of Orthopedics Surgery, Xijing Hospital, Fourth Military Medical University, Xi’an, Shaanxi People’s Republic of China; 3 Department of Stomatology, PLA Xizang Military Region General Hospital, Lhasa, Tibet People’s Republic of China; 4 Department of Stomatology, PLA 301th Hospital, Beijing, People’s Republic of China

Correction to: *Scientific Reports* 10.1038/srep40088, published online 05 January 2017

This Article contains an error. In Figure 1C, the incorrect image was used for PLSCSs after 2 hours. The correct version of Figure 1 appears below.

**Figure 1:**
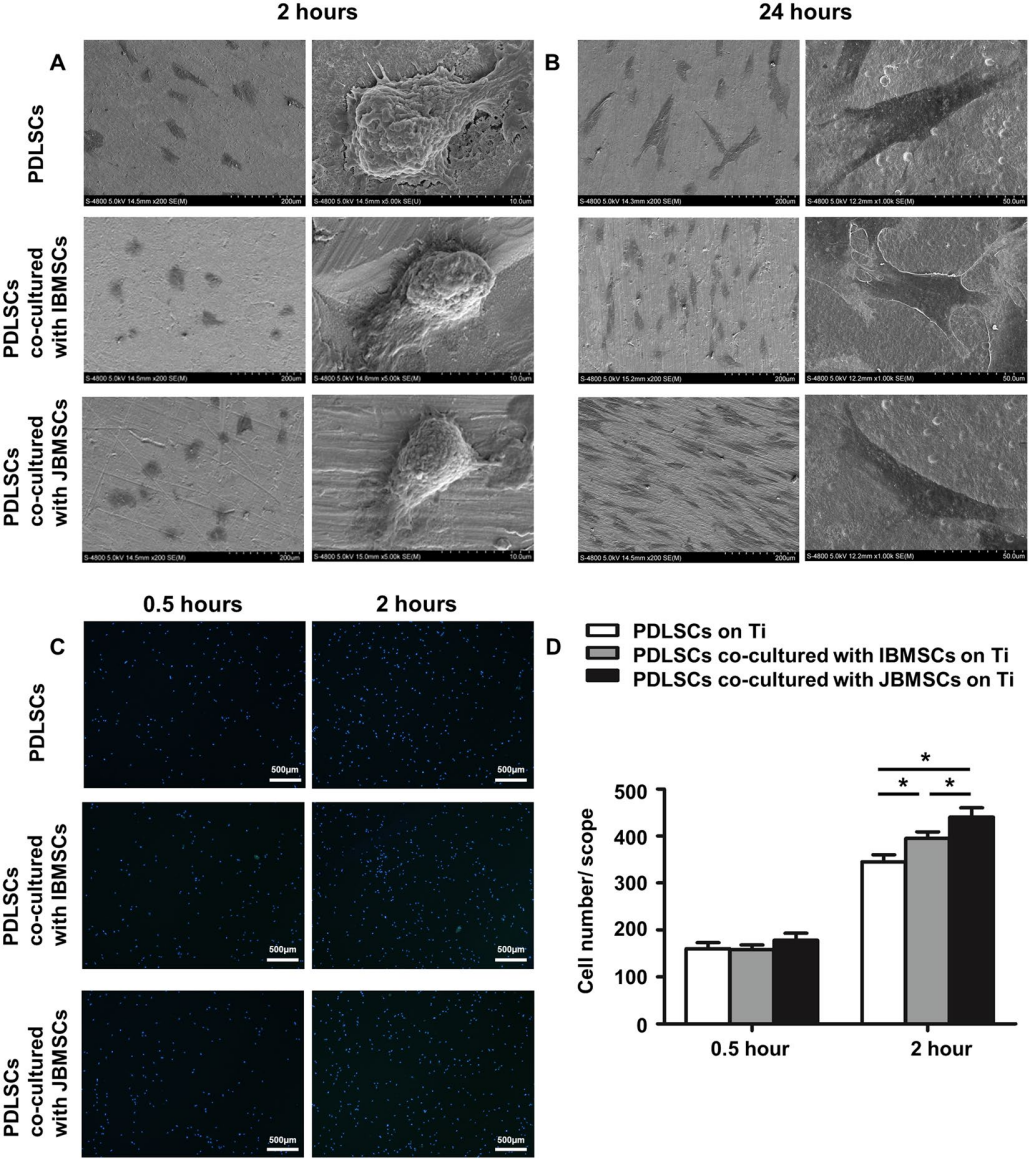
.

